# Hand-carried ultrasound performed at bedside in cardiology inpatient setting – a comparative study with comprehensive echocardiography

**DOI:** 10.1186/1476-7120-2-24

**Published:** 2004-11-17

**Authors:** Jeane M Tsutsui, Raquel R Maciel, Joicely M Costa, Jose L Andrade, Jose F Ramires, Wilson Mathias

**Affiliations:** 1 Echocardiography Laboratory of the Heart Institute (InCor) – University of Sao Paulo Medical School, Sao Paulo, Brazil; 2 Clinical Division of the Heart Institute (InCor) – University of Sao Paulo Medical School, Sao Paulo, Brazil

**Keywords:** Hand-carried ultrasound, comprehensive echocardiography, inpatient setting

## Abstract

**Background:**

Hand-carried ultrasound (HCU) devices have been demonstrated to improve the diagnosis of cardiac diseases over physical examination, and have the potential to broaden the versatility in ultrasound application. The role of these devices in the assessment of hospitalized patients is not completely established. In this study we sought to perform a direct comparison between bedside evaluation using HCU and comprehensive echocardiography (CE), in cardiology inpatient setting.

**Methods:**

We studied 44 consecutive patients (mean age 54 ± 18 years, 25 men) who underwent bedside echocardiography using HCU and CE. HCU was performed by a cardiologist with level-2 training in the performance and interpretation of echocardiography, using two-dimensional imaging, color Doppler, and simple calliper measurements. CE was performed by an experienced echocardiographer (level-3 training) and considered as the gold standard.

**Results:**

There were no significant differences in cardiac chamber dimensions and left ventricular ejection fraction determined by the two techniques. The agreement between HCU and CE for the detection of segmental wall motion abnormalities was 83% (Kappa = 0.58). There was good agreement for detecting significant mitral valve regurgitation (Kappa = 0.85), aortic regurgitation (kappa = 0.89), and tricuspid regurgitation (Kappa = 0.74). A complete evaluation of patients with stenotic and prosthetic dysfunctional valves, as well as pulmonary hypertension, was not possible using HCU due to its technical limitations in determining hemodynamic parameters.

**Conclusion:**

Bedside evaluation using HCU is helpful for assessing cardiac chamber dimensions, left ventricular global and segmental function, and significant valvular regurgitation. However, it has limitations regarding hemodynamic assessment, an important issue in the cardiology inpatient setting.

## Introduction

Bedside echocardiography can bring important anatomical and hemodynamic information for the management of critically ill patients, and is often required in hospitalized patients for the assessment of left ventricular function. Standard echocardiographic equipments, while optimal, have large size and sometimes are difficult to maneuver in the emergency room or intensive care units. Recently, hand-carried ultrasound (HCU) devices have been demonstrated to broaden the versatility in ultrasound application. Due to their portability and low cost, HCU acts like a stethoscope, providing information beyond physical examination at the point-of-care [1,2]. Although it has been shown to improve the detection of cardiovascular abnormalities over the physical examination, its role in the assessment of hospitalized patients is not completely established [3,4]. This study was undertaken to compare the findings of the bedside evaluation using HCU to the comprehensive echocardiography (CE), in cardiology inpatient setting.

## Methods

### Patients

We studied 44 consecutive hospitalized patients with cardiovascular disorders. Patients were included in the study when their referring physicians asked for a bedside evaluation with conventional echocardiography. In all patients, we performed the echocardiography with both HCU and CE within a maximal interval of 24 hours. The clinical characteristics of patient population are shown in Table 1. Among these patients, 61% were in the cardiac ward, 27% in the emergency room, and 12% in the intensive care unit. This study was approved by our Institutional Ethical Committee and informed consent was obtained from all participants or their legal representatives.

**Table 1:** Clinical characteristics

Variables	
Age (years)	54 ± 18
Male gender	25 (57%)
Cardiomyopathy	16 (36%)
Acute coronary syndrome	10 (23%)
Postoperative of cardiac surgery	9 (21%)
Valvulopathy	5 (11%)
Cardiogenic shock	3 (7%)
Total atrioventricular conduction block	1 (2%)

Data are mean ± SD or number (%) of patients.

### Study Protocol

All patients underwent two echocardiographic evaluations. First, HCU was performed with the portable device OptiGo (Philips Medical Systems, Andover, Massachusetts, USA) and, consecutively, by a commercially available system (HDI 5000, Philips Medical Systems, Bothell, Washington, USA) equipped with a 4-2 MHz transducer and second-harmonic imaging. HCU was performed by one same cardiologist with level 2-training in the performance and interpretation of echocardiography according to the specifications of the American Society of Echocardiography [5], after a period of instructions about the HCU settings. CE was performed by one experienced echocardiographer with level-3 training [5], and was considered the gold standard. Each investigator was blinded to the results of the other examination. The final echocardiographic report was based on the results of CE.

### Imaging Analysis

OptiGo is equipped with a 2.5 MHz phased-array transducer and operates on a rechargeable Lithium ion battery, which facilitates its use at bedside. HCU was performed using two-dimensional imaging, color Doppler flow mapping, and simple caliper measurements. Images were frozen and scrolled for review and the measurements were performed on-line.

CE evaluation included two-dimensional with second-harmonic imaging, M-mode, and both spectral and color Doppler flow mapping. Images were recorded on videotape or digitalized. The aorta, left atrium, left ventricular end-diastolic and end-systolic diameters, as well as interventricular septal and posterior wall thickness were measured according to the recommendations of the American Society of Echocardiography [6]. The left ventricular ejection fraction (LVEF) was visually estimated, and a normal ventricular function was defined as LVEF ≥ 55%. According to the segmental wall motion analysis, patients were divided as those with segmental wall motion abnormality (WMA) and those without WMA. Pericardial effusions were classified as mild, moderate, or large effusion.

Valve structure and function were analyzed. Dysfunctions were classified into mild, moderate, or severe degree according to the qualitative evaluation by HCU, and using both qualitative and quantitative parameters by CE [7]. A significant valvular regurgitation was defined in our study as those of moderate or severe degree [8]. On the other hand, non-significant valvular regurgitation was defined as those of no, trace, or mild degree. The estimation of transvalvar gradients and valvular areas in patients with prosthetic and stenotic valves, as well as the estimation of pulmonary artery pressure, were performed only by CE [7].

The intraobserver variability of CE findings was assessed in 15 randomly assigned patients, with analysis made at least 4 weeks apart. The interobserver agreement between the experienced echocardiographer and the level 2-trained cardiologist was assessed by the analysis of 15 CE recorded examinations.

### Statistical analysis

Continuous data are expressed as mean ± one standard deviation (SD) and categorical data as proportions. Comparisons between groups for continuous variables were made using Student *t *test. Chi-square and Fisher Exact tests were used for categorical variables. Agreement between HCU and CE results were assessed by the Kappa statistics. Interobserver and interobserver variability was determined by intra-class correlation and linear regression. A two-tailed p value <0.05 was considered significant.

## Results

**Figure 1:**
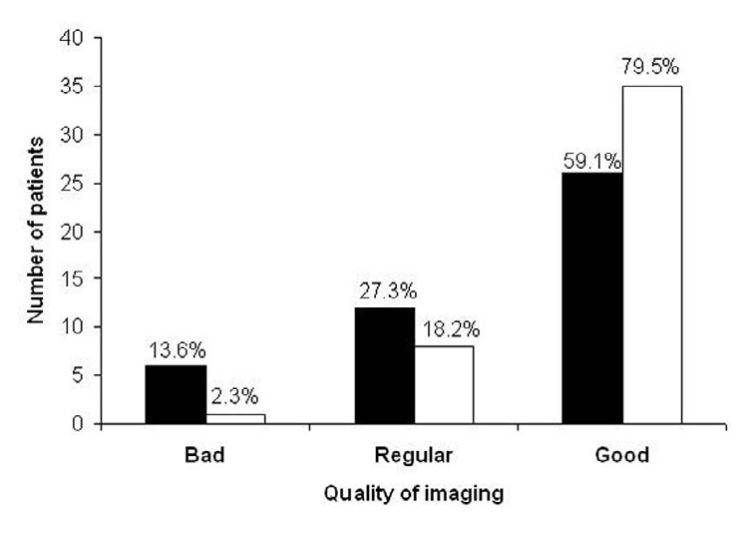
Image quality by hand-carried ultrasound and comprehensive echocardiography. Image quality obtained by hand-carried ultrasound device (solid bars) and by comprehensive echocardiography with second-harmonic imaging (open bars). Comprehensive echocardiography had a higher percentage of patients with good quality than hand-carried ultrasound. * p < 0.05 between groups.

Imaging analysis was feasible with HCU and CE in all patients. The percentage of patients with good, regular and bad image quality using CE were 80%, 18% and 2%, respectively. HCU had a lower percentage of patients with good image quality (59%), and a higher percentage of patients with regular (27%) and bad (14%) image quality (p < 0.05 versus CE), as shown in Figure 1.

### Determination of cardiac chamber dimensions and left ventricular function

There were no significant differences in cardiac chamber dimensions obtained by HCU and CE, except for the posterior wall thickness, which was lower when assessed by HCU (Table 2). Among the 44 studied patients, 24 (55%) had some degree of left ventricular dysfunction and 20 (45%) had normal left ventricular function. There was no difference between the LVEF estimated by CE and by HCU (Table 2).

**Table 2:** Cardiac chamber measurements obtained by comprehensive echocardiography (CE) and by hand-carried ultrasound (HCU)

Variables	CE	HCU
Aorta (mm)	28.7 ± 4.1	27.8 ± 4.2
Left atrium (mm)	45.5 ± 7.7	44.0 ± 7.5
LVED (mm)	57.1 ± 11.2	54.9 ± 10.7
LVES (mm)	44.4 ± 15.5	42.8 ± 10.7
IVST (mm)	10.0 ± 2.4	9.4 ± 2.4
PWT (mm)	9.6 ± 1.8	8.7 ± 1.7*
LVEF (%)	47 ± 16	44 ± 15

Data are mean ± SD. IVST = interventricular septal thickness; LVED = left ventricular end-diastolic diameter; LFEF = left ventricular ejection fraction; LVES = left ventricular end-systolic diameter; PWT = posterior wall thickness. * p < 0.05 compared to CE.

The analysis of segmental wall motion was not possible by HCU in two patients, due to poor endocardial border delineation, and was deemed feasible in all patients using CE. In the remaining 42 patients, HCU correctly identified eight of the 11 patients with WMA by CE, and failed to identify three of them. These three false-negative results occurred in patients with bad image quality by HCU. On the other hand, among the 31 patients without WMA by CE, HCU correctly identified 27 patients, and had four false-positive results. Among these four cases, three patients had global left ventricular dysfunction and one had asynchronic movement of the interventricular septum. The agreement between HCU and CE for the detection of WMA was 83% (Kappa = 0.58; p < 0.001).

CE identified nine patients with pericardial effusion, one patient with a moderate effusion and the remaining eight patients with mild effusions. HCU correctly detected and classified pericardial effusion in seven patients, and failed to diagnose two patients with mild effusions, both localized posterior to the left ventricle.

### Analysis of valvular dysfunction

Significant mitral valve regurgitation was diagnosed by CE in 16 patients, and by HCU in 13 of them. The agreement between HCU and CE for detection of this abnormality was 93% (Kappa = 0.85; p < 0.001). Significant aortic valve regurgitation was detected in three patients by CE and in two by HCU, and the agreement between the two techniques was 98% (Kappa = 0.89; p < 0.001). Significant tricuspid regurgitation was detected by CE in 16 patients and by HCU in 12 of them. However, there was one additional case misdiagnosed by HCU as of significant degree. The agreement between HCU and CE for the detection of significant tricuspid regurgitation was 88% (Kappa = 0.74; p < 0.001).

Four patients (9%) had aortic valve stenosis, two cases of moderate, one of mild, and the other one of severe degree. Qualitative analysis of valve structure by HCU was capable to identify aortic stenosis in two of these patients, but the severity of these lesions was not determined. There were six (14%) patients with prosthetic valves and eight (18%) patients with pulmonary hypertension, in which estimation of transvalvar gradients or valvular areas were performed only by CE. A complete evaluation of these patients was not possible using HCU due to its technical limitations in determining hemodynamic parameters.

### Intra and interobserver variability

The intraobserver agreement for detection of pericardial effusion, WMA, and significant valvular dysfunction was 100%, and the interobserver agreement was 91%. There was an excellent correlation between LVEF estimated in the first and second evaluation by the same observer (r = 0.91; p < 0.05). The correlation between LVEF estimated by the experienced echocardiographer and the cardiologist with level 2-training in echocardiography was r = 0.88 (p < 0.05).

## Discussion

The present study describes the value of bedside evaluation of cardiac patients using HCU in comparison to CE. Our results demonstrate that HCU may be used for the assessment of cardiac chamber dimensions, estimation of left ventricular function, and detection of WMA. Moreover, we found a good agreement between HCU and CE for detecting significant valvular regurgitation. However, HCU presents important limitations regarding the assessment of prosthetic and stenotic valves, as well as for the evaluation of patients with pulmonary hypertension, due to the lack of spectral Doppler.

Bedside echocardiography is a frequently used diagnostic tool in the cardiology inpatient setting and can affect the patient management, direct further diagnostic work-up, and modify therapeutic decisions. The recent development of portable ultrasound devices has the potential to allow quick and easy-to-use echocardiography at the point-of-care, although its value in hospitalized patients was not completely defined. In patients with cardiovascular disorders, HCU has been shown to increase the diagnostic accuracy over physical examination when performed by cardiologists with level-2 training in echocardiography [1]. In the present study we confirmed the usefulness of these portable devices for estimating cardiac chamber dimensions and left ventricular function, which are frequent indications for bedside echocardiographic examination. We also demonstrated a good agreement between CE and HCU for detecting WMA. We would like to emphasize that CE with second-harmonic imaging was chosen as the gold standard for evaluation of segmental wall motion, since it has already been proven that this imaging modality ameliorates the endocardial border delineation [9,10]. Another point to be noted is that our population included a high proportion of patients with left ventricular global dysfunction, in whom detection of segmental abnormality can be somehow challenging by non-experienced observers. In at least three of the four false-positive results, the presence of global myocardial impairment due to cardiomyopathy could lead to a confounding diagnosis of segmental impairment. In the false-negative cases, the use of second-harmonic modality improved the quality of imaging, allowing for a better visualization of endocardial thickening and detection of segmental left ventricular dysfunction. Our results are in accordance with recently published data demonstrating that HCU was highly concordant with clinical diagnosis of acute coronary syndrome based on the analysis of wall motion by these portable devices [11].

However, when considering the full spectrum of abnormalities in hospitalized patients, previous reports demonstrated that hand-carried bedside echocardiography failed to quantify valvular regurgitation and also missed findings relevant to clinical questions in a significant number of patients. These studies concluded that HCU falls far short of standard echocardiography in evaluation of critically ill patients [3,12]. We do believe that the lack of spectral Doppler in HCU is an important limitation for evaluation of cardiac patients, since this technique provides unique hemodynamic information, especially in patients with prosthetic or stenotic valves, and pulmonary hypertension. In our study population, these clinical conditions occurred in a considerable number of cases, since we included patients in the pre and postoperative period of valvular surgery.

### Limitations

In the present study, we did not evaluate the effect of HCU on patient management. Agreement between CE and HCU for detection of WMA was analyzed in 42 patients, since two patients were initially excluded because of inadequate acoustic window. The specific issue of agreement between HCU and CE according to image quality was not addressed in the present study because of the limited number of patients in each group.

## Conclusions and clinical implications

We concluded that HCU is useful for bedside assessment of left ventricular global and segmental left ventricular function as well as for evaluation of significant valvular regurgitation. However, it has limitations regarding hemodynamic assessment, which is an important issue in the cardiology inpatient setting.

Therefore, we emphasize that bedside echocardiography using HCU should be performed by cardiologist with at least level 2-training in echocardiography, and always complemented with CE when hemodynamic evaluation is necessary.

## Competing Interests

The authors declare that they have no competing interests.

## Authors' contributions

JMT and WMJ designed the study, did the selection and recruitment of the patients, and wrote the text. RRM and JMC performed the echocardiograms and participated in the subsequent analysis of data. JLA and JFR analyzed the statistics and revised this manuscript.
